# The role of a new CD44st in increasing the invasion capability of the human breast cancer cell line MCF-7

**DOI:** 10.1186/1471-2407-11-290

**Published:** 2011-07-12

**Authors:** Xin Jian Fang, Hua Jiang, Xv Peng Zhao, Wei Mei Jiang

**Affiliations:** 1 Department of Medical Oncology, The second People's Hospital of Lianyungang (Lianyungang Hospital affiliated to Bengbu medical college), No. 41, Hailian east Road, Lianyungang, Jiangsu 222000, The People's Republic of China; 2 Department of Emergency surgery, The fourth affiliated hospital of Chinese Medical Sciences University. No. 4, Chongshan east Road, Shen Yang 110032, The People's Republic of China

## Abstract

**Background:**

CD44, a hyaluronan (HA) receptor, is a multistructural and multifunctional cell surface molecule involved in cell proliferation, cell differentiation, cell migration, angiogenesis, presentation of cytokines, chemokines and growth factors to the corresponding receptors, and docking of proteases at the cell membrane, as well as in signaling for cell survival. The CD44 gene contains 20 exons that are alternatively spliced, giving rise to many CD44 isoforms, perhaps including tumor-specific sequences.

**Methods:**

Reverse transcriptase polymerase chain reaction (RT-PCR) and Western blotting were used to detect CD44st mRNA and CD44 protein in sensitive MCF-7, Lovo, K562 and HL-60 cell lines as well as their parental counterparts, respectively. The full length cDNA encoding CD44st was obtained from the total RNA isolated from MCF-7/Adr cells by RT-PCR, and subcloned into the pMD19-T vector. The CD44st gene sequence and open reading frame were confirmed by restriction enzyme analysis and nucleotide sequencing, and then inserted into the eukaryotic expression vector pcDNA3.1. The pcDNA3.1-CD44st was transfected into MCF-7 cells using Lipofectamine. After transfection, the positive clones were obtained by G418 screening. The changes of the MMP-2 and MMP-9 genes and protein levels were detected by RT-PCR and gelatin zymography, respectively. The number of the cells penetrating through the artificial matrix membrane in each group (MCF-7, MCF-7+HA, MCF-7/neo, MCF-7/neo+HA, MCF-7/CD44st, MCF-7/CD44st+HA and MCF-7/CD44st+Anti-CD44+HA) was counted to compare the change of the invasion capability regulated by the CD44st. Erk and P-Erk were investigated by Western blotting to approach the molecular mechanisms of MMP-2 and MMP-9 expression regulated by the CD44st.

**Results:**

Sensitive MCF-7, Lovo, K562 and HL-60 cells did not contain CD44st mRNA and CD44 protein. In contrast, the multidrug resistance MCF-7/Adr, Lovo/Adr, K562/Adr and HL-60/Adr cells expressed CD44st mRNA and CD44 protein. The CD44st mRNA gene sequence was successfully cloned into the recombinant vector pcDNA3.1 and identified by the two restriction enzymes. It was confirmed that the reconstructed plasmid contained the gene sequence of CD44st that was composed of exons 1 to 4, 16 to 17, and 1 to 205 bases of exons 18. The new gene sequence was sent to NCBI for publication, and obtained the registration number FJ216964. The up-regulated level of the mRNA of the CD44 gene and the CD44 protein were detected, respectively, by RT-PCR and flow cytometry in MCF-7 cells transfected with pcDNA3.1-CD44st. The invasiveness of the cells and the activity of MMP-2 and MMP-9 were clearly activated by HA treatment, and blocked by CD44 neutralizing antibody. MCF-7/CD44st cells pretreated with the neutralizing antibody against CD44, and the inhibitor of MAPKs signaling pathway, could strongly block the expression of P-Erk.

**Conclusions:**

A new CD44st was expressed in multidrug resistant MCF-7/Adr, Lovo/Adr, K562/Adr and HL-60/Adr cells. The expression vector pcDNA3.1-CD44st was cloned and constructed successfully, and stably transfected into MCF-7 cells. HA could interact with the new CD44st and regulate the expression of MMP-2 and MMP-9, which could increase the invasion capability of MCF-7 cells through the Ras/MAPK signaling pathway.

## Background

Tumor invasion is one of the major factors contributing to patient mortality during disease progression. When a tumor cell metastasizes, it initially penetrates the surroundings of the extracellular matrix (ECM), invades the vascular system, and transports to distant sites of the body [1].

**Figure 1:**
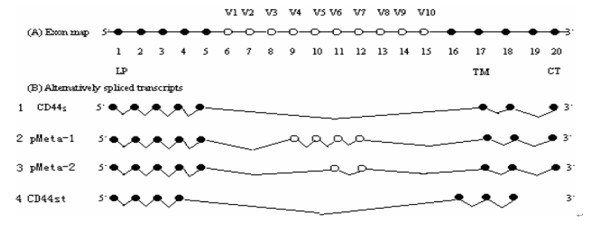
**The new CD44st mRNA and other CD44 isoforms.** The filled circles represent constant regions. The areas circled represent exons selected for splicing that potentially give rise to many variable isoforms. The cytoplasmic domain of CD44 may bond to the cytoskeleton. The central region of the CD44 molecule mediates lymphocyte homing and the amino-terminal region links to the HA. The cytoplasmic domain, the transmembrane region, and HA binding region are highly conserved.[9-10, 19]. B) (1) Standard CD44, which lacks the entire variable region. (2) pMeta-1; Exons v4 to v7 were inserted in turn into the intermediate part of exons 5 and 17. (3) pMeta-2; Exons v6 and v7 were interposed between exons 5 and 17 [4]. DNA sequencing confirmed that the reconstructed plasmid contained the sequence of the CD44st gene, which was composed of exons 1 to 4, exons 16 to 17, and 1 to 205 bases of 20 exons (Figure 1). The new gene sequence was sent to the National Center for Biotechnology Information (NCBI) for publication and registered with the number FJ216964.

The CD44 gene, which is located on human chromosome 11p, contains 20 exons and spans 50 kb. There are four distinct and characteristic regions in the CD44 protein: the leader peptide-encoding exon (exons 1-5) LP, the juxtamembranous extracellular variable domain (exons 6-14), the transmembrane-encoding exon (exon 17) TM, and the cytoplasmic domain (exons 18-20) CT [2,3] (Figure 1). By selective splicing, the cell-surface glycoprotein CD44 can theoretically generate approximately 800 isoforms [4].

Currently, dozens of CD44 isoforms have been discovered. The standard CD44 (CD44s) is the most common form, in which exon 5 is directly connected to exon 16, and lacks the entire variant exon region [1]. In our study, we used MCF-7/Adr cells to clone the novel CD44st, which contains exons 1 to 4, exons 16 to 17, and 1 to 205 bp of 18 exons (Figure 1). We found that HA-CD44st signaling leads to activation of MMP-2 and MMP-9 secretion in the MCF-7/CD44st cells, and subsequently increases a tumors' invasion capability [5,6].

The type I transmembrane glycoprotein receptor CD44 is a cell membrane receptor that links hyaluronate to the cytoskeleton ankyrin to mediate signal transduction [7]. CD44 also plays a role in cell migration, differentiation, and survival signaling, which is important both to normal cells and cancer cells. An animal model experiment has demonstrated that blocking CD44 with antibodies and antisense oligonucleotides decreases the malignant activities of the tumor [8]. The intracellular region of short tail CD44 (CD44st) contains exons 18 and 20, which is potentially linked to the ability of cytoskeleton protein (ankyrin and actin) to mediate signal transduction [9,10]. However, whether short tail CD44 (CD44st) is involved in signal transduction is currently unclear. Jiang H found that CD44st was not able to mediate the endocytosis process of the HA based on the fact that CD44st does not help HA integrate on the cell membrane [11].

HA, which has a high molecular weight, is widely distributed in the extracellular matrix (ECM), and could potentially combine with CD44 and participate in cell migration and cancer progression [12]. HA plays a role in anti-angiogenesis, but the degradation products of HA stimulate the generation of endothelial cells, angiogenesis and migration, resulting in CD44 receptor activation [13]. It has been previously demonstrated that HA synthase-1 may facilitate bladder cancer metastasis and therefore could potentially serve as a diagnostic marker [14].

Matrix metalloproteinases (MMP_S_) are zinc-required matrix-degrading enzymes that play an important role in tumor cell metastasis [15]. Recent studies have shown that hyalurinan strongly activates MMP-2 secretion; the principal reason is due to CD44 expression [16]. It has been demonstrated that MMP-9-CD44-EGFR interaction and the dimerization of the MMP-9 hemopexin domain are critical to the migration of fibrosarcoma cells [17]. Thus, it appears that HA, CD44, and MMP_S _are all involved in the initiation of tumor invasion and are attractive targets for tumor therapy.

## Methods

### Reagents and Cell Culture

Fetal bovine serum was purchased from Hangzhou Sijiqing Biological Engineering Materials Co, Ltd. The following reagents used in this study were purchased from Invitrogen: 1640 medium, 2.5% trypsase with EDTA, Trizol, opti-MEM-I Reduced Serum Medium (OPTI-MEM), Lipofectamine 2000 and G418. The RT-PCR kit was purchased from Fermentas. Hyaluronan was purchased from Sigma. Matrigel was purchased from Becton Dickinson and Company. Transwell was purchased from Corning, and the FITC labeled CD44 antibody used for detection of flow cytometry was purchased from eBioscience. The CD44 antibody used for western blot detection was purchased from Abcam. The CD44 blocking antibody was obtained from Neromarker. Manumycin A, PD98059, Phospho-p44/42 MAPK (Thr202/Tyr204) Mouse mAb, and p42 MAP Kinase (Erk) Antibody were purchased from Cells singnal technology. The recombinant vector pcDNA3.1 was generously supplied by Dr. XU Wen-rong in the medical college of Jiangsu University. Human breast cancer cell lines (MCF-7 and MDR derivative MCF-7/Adr) and human colorectal cancer cell lines (Lovo and MDR derivative Lovo/Adr), human promyelocytic leukemia cell lines (HL-60 and MDR derivative HL-60/Adr), and human chronic myeloid leukemic cell lines (K562 and MDR derivative K562/Adr) were all obtained from the Shang-hai cell repository of Academia Sinica and the cells were maintained in 1640 medium with 10% heat-inactivated FBS at 37°C in a humidified atmosphere containing 5% CO_2_.

### Construction and expression of eukaryotic expression vector pcDNA3.1-CD44st, RNA extraction, and reverse transcription-PCR

The total RNA of the cultured cells was extracted using Trizol reagent according to the manufacturer's directions. The RT-PCR kit was used to carry out RT-PCR of each RNA template according to the manufacturer's instructions. In brief, reverse transcription of each mRNA transcript occurred during incubation for 5 minute at 70°C, followed by incubation for 1 hour at 42°C and finally at 70°C for 10 minute. The primers of the CD44 (GenBank.NO.AJ251595) are as follows: forward primer, 5'-GGATGGACAAGTTTTGGTGGCACG-3'; reverse primer, 5'-GGTTACACCCCAATCTTCATGTCC-3'. PCR amplification for CD44st occurred as follows: incubation at 94°Cfor 5 minute, 30 cycles at 94°C for 30 seconds, incubation at 66°Cfor 30 seconds, and at 72°C for 2 minute, followed by incubation at 72°C for 10 minute, and storage at 4°C. The β-actin gene primers which were used as a control are as follows: forward primer, 5'-CTCGCGCTACTCTCTCTTTC-3', reverse primer, 5'-CATGTCTCGATCCCACTTAAC-3'. Reverse transcription occurred as above, and PCR amplification as follows: incubation at 94°C for 5 minute, 30 cycles at 94°C for 30 seconds, incubation at 58°C for 30 seconds, and at 72°C for 30 seconds, then at 72°C for 10 minutes, followed by storage at 4°C. PCR amplification for MMP-2 (GeneBank accession no. NM 004530) (forward primer, 5'-CGGTGCCCAAGAATAGATG-3; reverse primer, 5'-AAAGGAGAAGAGCCTGAAGTG-3') occurred as follows: incubation at 94°C for 5 minute, followed by 30 cycles at 94°C for 30 seconds, then incubation at 59.1°C for 30 seconds, and incubation at 72°C for 30 seconds, followed by incubation at 72°C for 10 minutes, and storage at 4°C. MMP-9 (GeneBank accession no. NM 004994) (forward primer, 5'-CGGAGCACGGAGACGGGTAT-3'; reverse primer 5'-GCCGCCACGAGGAAAACT-3') PCR amplification occurred as follows: incubation at 94°C for 5 minute, followed by 30 cycles at 94°C for 30 seconds, incubation at 62.7°C for 30 seconds, and at 72°C for 45 seconds, followed by incubation at 72°C for 10 minute, and storage at -20°C.

### Gene clone and Plasmid construction

**Figure 2:**
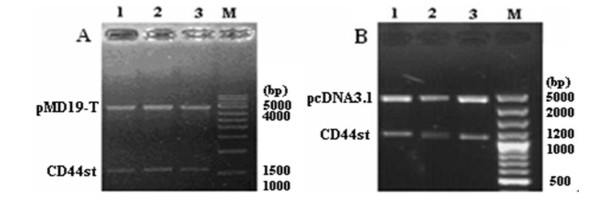
**Construction of recombinant plasmid and pcDNA3.1 vector.**. A) 1% Agarose DNA gel electrophoresis for three positive TA clone plasmids digested by restriction enzyme EcoR I and Kpn I; lane 1 to lane 3: three positive TA clone plasmids; lane M: DL-3000 marker. B) 1% Agarose DNA gelelectrophoresis for pcDNA3.1-CD44st digested with two restriction enzymes: EcoR I and Kpn I; lane 1 to lane 3: three positive plasmids, respectively; lane M: DL-10000 Marker.[9,10,19]. B) (1) Standard CD44, which lacks the entire variable region. (2) pMeta-1; Exons v4 to v7 were inserted in turn into the intermediate part of exons 5 and 17. (3) pMeta-2; Exons v6 and v7 were interposed between exons 5 and 17 [4]. DNA sequencing confirmed that the reconstructed plasmid contained the sequence of the CD44st gene, which was composed of exons 1 to 4, exons 16 to 17, and 1 to 205 bases of 20 exons (Figure 2). The new gene sequence was sent to the National Center for Biotechnology Information (NCBI) for publication and registered with the number FJ216964.

The CD44st mRNA extracted from MCF-7/Adr cells via reverse transcription-PCR was sub-cloned to synthesize the full-length human CD44st cDNA. The EcoRI and KpnI restriction sites were incorporated into the forward and reverse primers as follows: CD44st (EcoR I) forward primer 5'-GGGAATTCATGGACAAGTTTTGGTGGCACG-3', and CD44st (Kpn I) reverse primer 5'-GGGGTACCTTACACCCCAATCTTCATGTCC-3'. The GenBank accession number and product amplification protocol of CD44st mRNA are listed above. According to the manufacturers' instructions, the PCR products were visualized by ethidium bromide staining and subcloned into the pMD19-T vector (Figure 2A). Plasmid isolation from recombinant *E. coli *was carried out using Purelink Quick plasmid minipreps kit (Axygen) according to the manufacturers' instructions, and then double digested with EcoRI and KpnI restriction enzymes and confirmed by DNA sequencing. Next, the CD44st gene was inserted into the eukaryotic expression vector pcDNA3.1 (Figure 2B).

### Transfection and obtaining the positive clone

The pcDNA3.1 and the pcDNA3.1-CD44st vectors were transferred to MCF-7 cells according to the manufacturers' protocol. After incubation for 48 hours, the transfected cells were replanted and exposed to G418 containing medium (800 μg/ml) for 1 week. When a large number of the cells appeared to be dying, the concentration of G418 was reduced to 200 μg/ml and the cells were incubated for 2 weeks. The positive clones were identified and selected for further development, and then cultured with a 200 μg/ml G418 solution to maintain resistance. The expression of CD44st was confirmed by RT-PCR, DNA sequencing, and flow cytometric analysis.

### Flow cytometric analysis

Cells in the logarithmic phase were harvested via the cell scraper technique. After being washed three times with a PBS solution, the harvested cells were blown uniformly into PBS buffer to prepare a single-cell suspension. 5 × 10^5 ^cells/ml were incubated with the FITC conjugated rabbit antimouse antibody for 1 hour at 4°C, centrifuged at 1000 rpm for 5 min, and then the supernatant was discarded and cells were resuspended with 500 μL PBS. The relative fluorescence intensity was detected using the flow cytometer (Becton Dickinson). The data were analysed with Cell Quest software. The experiment was repeated three times.

### The effects of CD44st on the invasion ability of MCF-7 cells

#### Treatment of Cells

The cells were digested by 0.25% trypsin in combination with 0.02% EDTA, and then 1.5 × 10^6 ^cells were seeded into a new culture flask and diluted with 1.5 ml 1640 medium without heat-inactivated 10% FBS. The cells pretreated with CD44 blocking antibody were incubated for 3 hours with serum-free medium containing CD44 blocking antibody (20 μg/ml). HA (100 μg/ml) was added to the HA treatment groups and the cells were then incubated for 24 hours. In order to eliminate potential contamination by growth factor, the HA solution was boiled at 100°C for 5 minutes before use [18].

#### Invasion assays by Matrigel

The transwell method (Corning) was used to perform invasion assays. The pretreatment filters (8 μm pore-size, Matrigel 100 μg/cm^2^) were rehydrated with 100 μl of medium. 1 × 10^5 ^cells in the volume of 100 μl medium were seeded onto the upper part of each well. The volume of 1 ml serum-free 1640 medium containing 0.1% BSA was added to the lower compartment of the transwell. After incubation for 24 h at 37°C, the cells located upon the membranes were wiped with the cotton buds. The cells that had invaded the lower part of the transwell were stained with the gentian and counted using light microscopy (×200, 5 random fields). The invasion capability of the tumor cells was determined by averaging the number of positively stained cells in each of the microscopic fields.

#### Gelatin Zymography

Gelatin zymography analysis was performed on 20 μl culture medium per sample according to the manufacturers' protocol. The positive control was a 1:1 ratio of whole human blood and 2 × non-denaturing loading buffer.

#### Western Blot Analysis

3 × 10^5 ^MCF-7/CD44st cells were seeded in wells of a 6-well plate, grown under routine conditions, and harvested at 80-90% confluence. Each sample was resuspended in 60 μl of boiling 2 × loading buffer. The activation of ERK was detected with a specific antibody to the phosphorylated form of ERK. After cells were lysed by sonication, the cell lysate was subjected to centrifugation at 12,000 × *g *for 15 min at 4°C. The gels were electro-transferred using an electrophoresis apparatus (Bio-Rad). The total protein of 20 μl/lane was isolated using 10% SDS-PAGE and transferred to a nitrocellulose transfer membrane (0.2 μm). A 5% non-fat milk solution with TBST was used to block the membrane for 1 hour at room temperature. The membrane was incubated with primal mouse anti-human monoclonal antibody (1:500) at 4°C overnight, washed with eluant three times within 30 minutes, incubated with secondary goat anti-rat monoclonal antibody (1:2000) for 1 h at room temperature, and finally washed as above. Immunoblots were visualized using a DAB coloring reagent kit according to the manufacturers' protocol. Gelatin zymography was performed to determine the activity level of MMPs in the culture medium.

#### Statistical analysis

Data were all presented as mean ± standard deviation (SD). To test for statistical significance, experimental data were analysed by One-Way ANOVA and SNK-q test. A p value less than 0.05 was considered to indicate a statistically significant result. All of the experiments were repeated in triplicate.

## Results

### CD44st is expressed in multidrug resistant cells, but not in parental, sensitive cell lines

**Figure 3:**
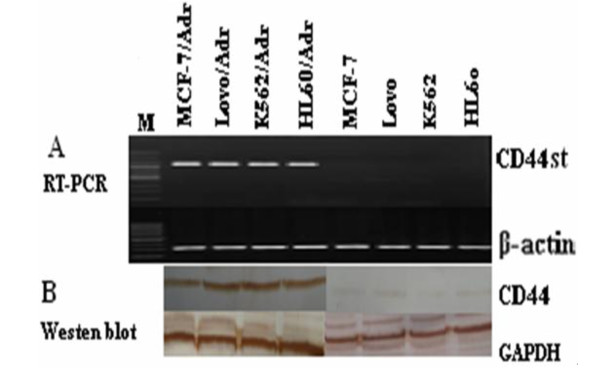
**CD44st differentially express in sensitive and MDR cancer cells.** A) The expression of CD44st in various human cancer cell lines. Total RNA from sensitive and drug-resistant cells was analyzed using CD44st semi-quantitative RT-PCR. The ß-actin gene was amplified using semi-quantitative RT-PCR and used as a control. RT-PCR products were run on a 1% agarose gel to show the expression levels of the CD44st gene and ß-actin gene. The CD44st gene in these cells was also tested by DNA sequencing. B) Cell lysates from sensitive and drug-resistant cells were immunoblotted with an anti-CD44 antibody.

We chose the (MDR) cell lines and their respective P-glycoprotein-negative, drug-sensitive, parental cell lines in order to study the relationship between CD44st expression and multidrug resistance by RT-PCR and Western blot analysis. As shown in Figure 3 A-B, both CD44st mRNA and CD44 protein were not expressed in the sensitive MCF-7, Lovo, K562, and HL-60 cell lines. In contrast, the corresponding multidrug resistant cell lines all expressed CD44st mRNA and CD44 protein. This result suggests that a functional link between CD44st expression and MDR may exist.

### Expression of CD44st gene and protein in MCF-7/CD44st cells

**Figure 4:**
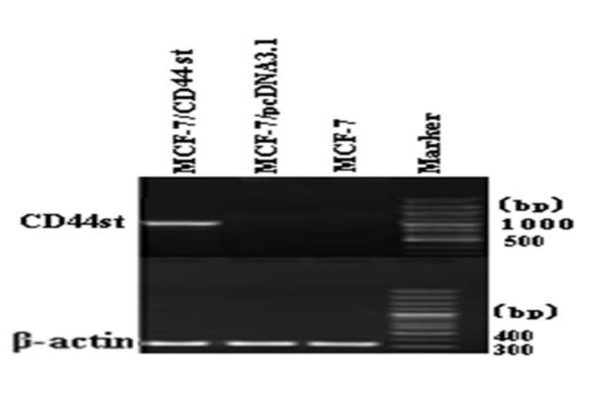
**Expression of CD44st in MCF-7, MCF-7/neo, and MCF-7/CD44st cells**. RT-PCR products were run on an agarose gel to indicate the expression levels of the CD44st gene and β-actin gene. The anticipated 1023-bp product for CD44st was present in MCF-7/CD44st cells. The β-actin gene was amplified using semi-quantitative RT-PCR and used as a loading control. The 330-bp product for β-actin gene expression was present in all cells.

**Figure 5:**
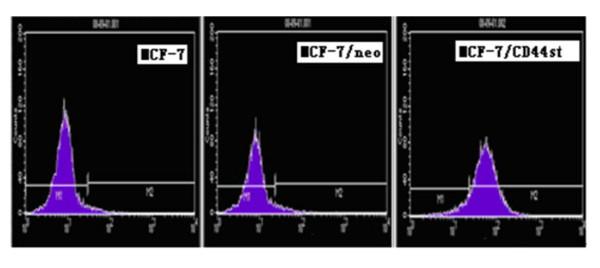
**Expression of the CD44 protein in MCF-7, MCF-7/neo, and MCF-7/CD44st cells**. Flow cytometric analysis was used to examine the expression level of CD44 protein in MCF-7, MCF-7/neo, and MCF-7/CD44st cells.

The expression of CD44st mRNA and CD44 protein in MCF-7/CD44st cells (MCF-7 cells transfected with pcDNA3.1-44st), MCF-7 cells, and MCF-7/neo cells (MCF-7 cells transfected with pcDNA3.1) were detected using RT-PCR and flow cytometry analysis, respectively. Among these cell lines, only MCF-7/CD44st cells expressed a high level of CD44st mRNA transcripts and CD44 protein (Figure 4, 5). As shown in Figure 5, the mean percentage of CD44 protein expressed was (8.87 ± 1.5)% in MCF-7 cells, (8.17 ± 1.2)%in MCF-7/neo cells, and (69.8 ± 2.3)% in MCF-7/CD44st cells. There were no major differences between MCF-7 and MCF-7/neo cells regarding CD44 expression. We then compared MCF-7/CD44st cells with MCF-7 or MCF-7/neo cells, and statistical analysis demonstrated significant differences in CD44 expression (P < 0.01). This result indicated that the cells transfected stablely with pcDNA3.1-CD44st were constructed successfully.

### MMP-2 and MMP-9 gene expression in MCF-7/CD44st cells pretreated with HA

**Figure 6:**
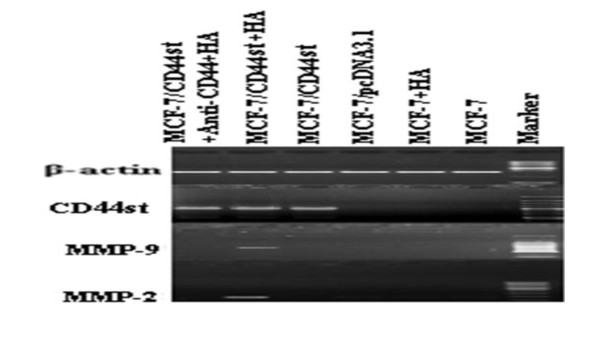
**Expression of CD44st and expression of HA inducting MMP-2, MMP-9 mRNA**. Cells treated with HA were cultured in serum free medium containing or without HA (100 μg/ml) for 24 h. The expression levels of MMP-2 and MMP-9 mRNA were measured by RT-PCR.

**Figure 7:**
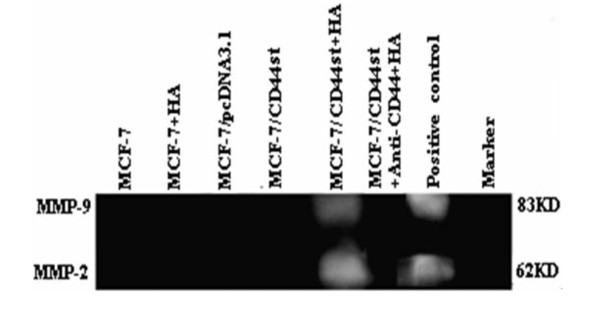
**Secretion of HA inducting MMP-2 and MMP-9 protein**. Cells treated with HA were incubated in serum free medium with or without HA (100 μg/ml) for 24 h. The secretion of HA inducting MMP-2 and MMP-9 was detected by zymography. Normal human blood was used as a positive control to identify MMP-9 and MMP-2 on gelatin zymograms.

Using MCF-7/CD44st cells, we next examined the effect of HA treatment on MMP-2 and MMP-9 secretion. Cells were incubated in serum-free medium for 3 h in the presence or absence of HA. After incubation for 24 h, cells in each group were collected and the expression levels of interest were detected using RT-PCR. Meanwhile, the culture medium was examined with zymography. As is shown in Figure 6 and 7, MCF-7/CD44st cells secreted MMP-2 and MMP-9 in the presence of HA, and HA pretreatment activated the secretion, whereas the other groups did not secrete detectable amounts of MMP-2 and MMP-9 nor respond to the HA treatment. These results suggest that HA and CD44st not only induce an increase in the secretion of MMP-2 and MMP-9, but they also are involved in MMP-2 and MMP-9 activation in MCF-7/CD44st cells. Furthermore, it is noteworthy that the CD44 blocking antibody was able to prevent MMP-2 and MMP-9 secretion in MCF-7/CD44st cells.

### CD44st and hyaluronan interaction causes increase of invasion in MCF-7/CD44st cells

**Figure 8:**
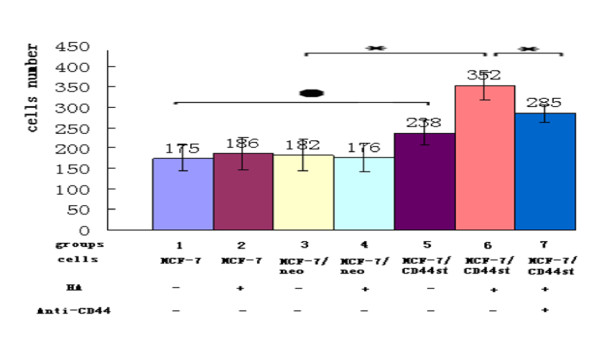
**CD44st and hyaluronan interaction leads to an increase in MCF-7/CD44st invasion capabilities**. The invasive capability of the tumor cells was assayed in the presence (+) and absence (-) of HA (100 μg/ml) for 24 h with transwell pretreated filters. Cells in the pretreatment with CD44 blocking antibody groups were pretreated with anti-CD44 (20 μg/ml) for 3 h, and then treated with HA (100 μg/ml) for 24 h. ● P > 0.05, *P < 0.05.

A transwell experiment with pretreatment filters was used to study the influence of the expression of CD44st on the invasion of tumor cells. Each group was loaded onto the upper compartment of the transwell. Cells that penetrated the membranes were counted. As shown in Figure 8, the number of invasive cells in the MCF-7/CD44st+HA group occurred at a higher rate compared to the other experimental groups (p < 0.05). There were no statistically significant differences among MCF-7, MCF-7+HA, MCF-7/neo, MCF-7/neo+HA, and MCF-7/CD44st groups (p > 0.05). After MCF-7/CD44st cells were pretreated with a CD44 blocking antibody, the number of cells able to penetrate the membranes decreased (p < 0.05). This indicated that the invasiveness of MCF-7/CD44st cells was clearly activated after being treated with HA. Moreover, the invasiveness of MCF-7/CD44st cells was significantly suppressed by pretreatment with CD44 blocking antibody. Furthermore, there was no statistical difference in the number of the cells between the MCF-7 group and the groups pretreated with HA. These results suggest that the activation of tumor invasiveness relies on the link between HA and CD44st.

### Ras-MAPK signaling pathway plays a role in HA-induced MMP-2 and MMP-9 secretion in MCF-7/CD44st cells

**Figure 9:**
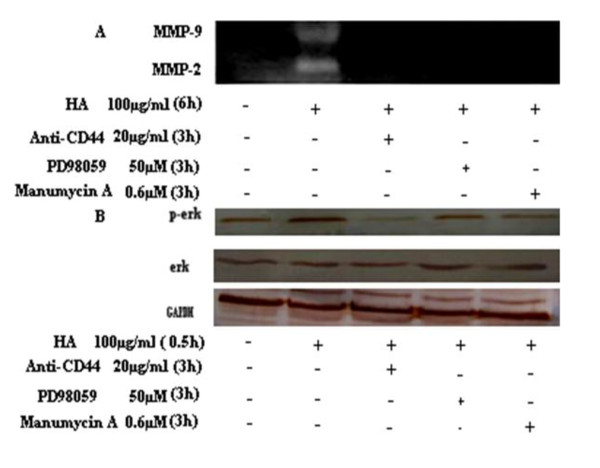
**Inhibition of MMP-2 and MMP-9 secretion and activation MAPK signaling pathway**. A) MCF-7/CD44st cells were pretreated (+) or unpretreated (-) with CD44 antibody, PD98059 and manumycin A, and then stimulated with HA (+) or without HA (-) for the indicated concentrations and time periods. Conditioned medium samples were harvested and subjected to zymography (top panel). B) MCF-7/CD44st cells were pretreated (+) or unpretreated (-) with CD44 antibody, PD98059 and manumycin A, for the indicated concentrations and time periods, and then incubated with 100 μg/ml of HA (+) or without HA (+) for 0.5 h. Cells lysates were subjected to immunoblotting with anti-phospho-ERK (bottom panel).

Previous evidence has suggested that the conjugation of the HA-CD44 receptor could activate the MAPK signaling pathway [18]. Therefore, we presumed that the novel alternative splicing clone CD44 may have a functional relationship with the pathway. Therefore, we studied the effect of the HA→CD44st→ras→erk signaling pathway on HA-induced MMP-2 and MMP-9 secretion. As is shown in Figure 9A, pretreatment of the cells with MAPK inhibitors (manumycin A and PD98059) significantly suppressed HA-induced MMP-2 and MMP-9 secretion. Moreover, we investigated the role of a CD44 blocking antibody regarding the expression of MMP-2 and MMP-9. As expected, after pretreatment with CD44 blocking antibody, MCF-7/CD44st cells exhibited significantly reduced MMP-2 and MMP-9 secretion, which was examined by gelatin zymogram analysis. These results clearly indicated that CD44st participation is necessary for secretion of MMP-2 and MMP-9 to be activated by HA. To verify this observation, we examined the expression of ERK and phosphorylated ERK in MCF-7/CD44st cells. Indeed, we found that HA stimulation up-regulated the phosphorylated ERK in MCF-7/CD44st cells. In contrast, ERK activation was significantly reduced in MCF-7/CD44st cells pretreated with CD44 blocking anti-bodies, manumycin A and PD98059 (Figure 9B). These results demonstrate that the CD44st→ras→MAPK pathway is involved in the HA-induced activation of MMP -2 and MMP-9 secretion.

## Discussion

In this study, we determined that CD44st mRNA transcripts and CD44 protein were highly expressed in the multidrug resistant cell line cells, but not in the parental sensitive cell lines. It appears that there must exist a functional link between CD44st and multidrug resistance. Additionally, we studied the function of the new CD44 isoforms (GeneBank NO. FJ216964) regarding tumor invasion using gelatin zymography and transwell experiments. Our findings indicated that the invasiveness of MCF-7/CD44st cells was clearly activated upon treatment with HA. Moreover, the invasiveness of MCF-7/CD44st cells was significantly suppressed by pretreatment with CD44 blocking antibody. Furthermore, statistically significant differences in the number of cells located in the lower compartment of the transwell experiment did not occur between the group of MCF-7 and the group of MCF-7 pretreated with HA. These results suggest that the activation of the tumor invasiveness is dependent upon HA linkage to CD44st. Finally, we approached the role of Ras-MAPK signaling pathways in HA-induced MMP-2 and MMP-9 secretion in MCF-7/CD44st cells. These results indicated that MMP-2 and MMP-9 secretion in MCF-7/CD44st cells is potentially regulated by the HA-CD44st signaling pathway. In addition, we demonstrated that CD44 blocking antibody could block MMP-2 and MMP-9 secretion as well as tumor invasion in MCF-7/CD44st cells.

The major causes of treatment failure in cancer are the development of metastases and drug resistance. Currently, there is some evidence indicating that metastases and multidrug resistance may have a functional link. The extracellular matrix metalloproteinase inducer (EMMPRIN) that is abundantly expressed on the surface of tumor cells potentially stimulates the production of matrix MMPs. MDR cells over-expressed EMMPRIN compared to the parental sensitive cell lines, and were capable of producing higher levels of MMP-1, MMP-2, and MMP-9 [20]. In mammary cancer cells, the interaction among HA, CD44, and EMMPRIN potentially regulates the localization and function of the plasma membrane transporter, as well as the tumor MDR [21]. CD44 and P-glycoprotein encoded by the MDR-1 gene have a functional link, and collectively regulate malignant biological phenotypes, tumor invasion, and MDR [22]. Similarly, some recent studies also indicate that both HA and CD44 are involved in chemotherapeutic drug resistance in many cancers [23-27].

During tumor progression, blockage of the HA-CD44 signaling pathway may provide a new target that overcomes multidrug resistance in breast and ovarian carcinomas [28]. The mitogen-activated protein kinase (MAPK) signaling pathway is important to normal cells as well as cancer cells. Inhibition of MAPK signaling pathways could suppress renal cell carcinoma growth by harming the tumor blood vessels [29]. HA-CD44 potentially mediates the activation of ankyrin and Rho GTPase as the tumor progresses, and thus are also important markers for early diagnosis and evaluation of disease prognosis [30].

The role of the expression of CD44 isoforms in tumor progression has been widely studied in the last decade. When CD44, HA, and heparanase are highly expressed in breast cancer cells, they generate a microenvironment that facilitates tumor progression and invasion [31]. The phenotype of CD44+/CD24^- ^in breast cancer cells exhibits a higher potential compared to other phenotypes of breast cancer cell lines, however, this is not sufficient evidence to elucidate the relationship with pulmonary metastasis [32]. However, it has been previously reported that the expression of CD44 potentially prevents breast cancer cells from metastasizing. In an experiment, the authors found that HA containing material could inhibit the invasion of the CD44 positive tumor cells. During cancer progression, HA-CD44 epithelial-stromal interaction potentially restrains tumor metastasis [33]. As a result, some arguments remain regarding the relationship between CD44 expression and tumor progression and metastasis.

## Conclusions

In summary, the results we present in this study suggest that the novel CD44st gene, cloned from MCF7-/Adr cells, plays an important role in the HA-dependent activation of MMP-2 and MMP-9, as well as the invasiveness of MCF-7/CD44st cells. Furthermore, it appears that the new CD44st gene and MDR-1 gene have some functional links which influence the tumor's multidrug resistance, invasion, and metastasis all together. This lay a ground work for further study. Our results potentially provide a new therapeutic target for tumor invasion through the regulation of the CD44st signal pathway. Finally, expression of CD44st could potentially be used as a dominant selectable marker for the detection of the phenotype of multidrug resistance and tumor cell invasion capabilities.

## Abbreviations

HA: hyaluronan. RT-PCR: reverse transcript polymerase chain reaction; ECM: extracellular matrix; EMMPRIN: extracellular matrix metalloproteinase inducer; MDR: multidrug resistance; MMP-1: matrix metalloproteinase-1; MMP-2: matrix metalloproteinase-2; MMP-9: matrix metalloproteinase-9; OPTI-MEM: opti-MEM-Reduced Serum Medium.

## Competing interests

The authors declare that they have no competing interests.

## Authors' contributions

FXJ contributed to the study design and experimental research. JH offered the fund to the experiment and contributed to the study design.

JWM contributed to the data analysis. All the authors were involved in drafting the manuscript and have given final approval of the version to be published.

## Pre-publication history

The pre-publication history for this paper can be accessed here:

http://www.biomedcentral.com/1471-2407/11/290/prepub
